# ‘Main the gap!’ The view of healthcare professionals on gains and pitfalls of traditional and innovative models for providing mental health care to imprisoned persons with a severe mental illness in Spain

**DOI:** 10.1192/j.eurpsy.2023.1004

**Published:** 2023-07-19

**Authors:** J. Antón Basanta, S. Paz Ruiz, V. P. Estévez Closas, Á. López López, L. F. Barrios, A. Calcedo-Barba

**Affiliations:** 1 Sociedad Española Sanidad Penitenciaria, Barcelona; 2 SmartWorking4U SLU, Benicassim; 3 Hospital Psiquiátrico Penitenciario, Fontcalent; 4 Universidad de Alicante, Alicante; 5 Sociedad Española Psiquiatría Legal, Madrid, Spain

## Abstract

**Introduction:**

Different mental health care provision models coexist in prisons in Spain. The Ministry of Interior applies a traditional model to secure mental health care to 83% of the country imprisoned population. Three autonomous regions with acquired competencies for health care provision (17% of the imprisoned population) are implementing innovative care models.

**Objectives:**

To explore the views of healthcare professionals on models of mental health care provision for imprisoned persons with a serious mental illness (SMI) in Spain.

**Methods:**

21 healthcare professionals (13 physicians, 5 nurses, 3 pharmacists) working in prisons, penitentiary psychiatric hospitals and a psychiatric in-prison unit took part in 5 online, 2 hours focus groups and one in-deep interview between 31^st^ May and 20^th^ July 2022. The moderator used open-ended questions to research into the characteristics of mental health care models and on the challenges for implementation. Focus groups were audiotape recorded and transcribed. Transcripts were analysed applying thematic analysis.

**Results:**

Healthcare professionals reported that within the traditional model of healthcare provision, the psychiatric care of SMI imprisoners relies on correctional general practitioner physicians (GP). Psychiatrists act as external care providers. There are two psychiatric penitentiary hospitals with a strong correctional character for in-hospital care. Acute psychiatric care happens in prisons or at the local general hospital. Healthcare records remain within the penitentiary organization and outside the accesible healthcare information system. In consequence, there is fragmentation and delocalization of mental health care. An innovative approach consists of a dedicated mental healthcare unit within the prison with continuous psychiatric supervision of imprisoners with SMI and good quality psychiatric care. Schizophrenia and hyperactive attention deficit disorder persons benefit the most. Continued mental health care in the community remains a challenge. Another model of care is centred in the SMI imprisoned person. Acute and rehabilitation psychiatric penitentiary units operate within a network of mental health and social care resources in the community, coordinated by a liaison nurse. Individualised care plans keep SMI persons in their social environment. Costs of implementation are high. Clear definition of roles; investment in dedicated staff and shared information systems are challenges to overcome.

**Conclusions:**

Innovative models of mental health care are needed to benefit imprisoned persons with a SMI in Spain. A decided national and regional will is paramount to overcome challenges.

**Disclosure of Interest:**

None Declared

